# An Interesting Case of Permanent His-Bundle Pacing and a Review of the Current Literature

**DOI:** 10.19102/icrm.2017.080404

**Published:** 2017-04-15

**Authors:** Fatima Ezzeddine, Gopi Dandamudi

**Affiliations:** ^1^Indiana University School of Medicine, Indianapolis, IN

**Keywords:** Biventricular pacing, His-bundle pacing, infranodal block, right ventricular pacing

## Abstract

Permanent His-bundle pacing (HBP) is a true physiological form of ventricular pacing that has been shown in recent years to be both safe and feasible in clinical practice. However, there are limited data about its long-term performance, especially when compared with both right ventricular and biventricular pacing. In this article, we present a thought-provoking case study that illustrates the usefulness of permanent HBP in a patient with long-standing complete infranodal heart block and progressive heart failure, and discuss the current literature highlighting the evidence behind this form of permanent pacing.

## Introduction

Right ventricular pacing (RVP) has been the standard of care in cardiac pacing for decades. However, a growing body of evidence has shown that RVP leads to dyssyn-chronous activation of the ventricles, resulting in adverse clinical outcomes such as increased incidence of heart failure (HF), atrial fibrillation (AF), mitral regurgitation, and mortality.^1–3^ Even though biventricular pacing (BiVP) has been shown to improve morbidity and mortality in patients with advanced HF and wide QRS durations, it has had more equivocal results in the rest of the population who require ventricular pacing.^4,5^ By reviving and maintaining the latent native conduction system, His-bundle pacing (HBP) can offer an attractive alternative to cardiac pacing that avoids the detrimental effects of cardiac dyssynchrony. Here, we present a case study to illustrate the usefulness of HBP from a clinical perspective, followed by a review of the current literature discussing this form of permanent pacing.

## Case presentation

**Figure 1:**
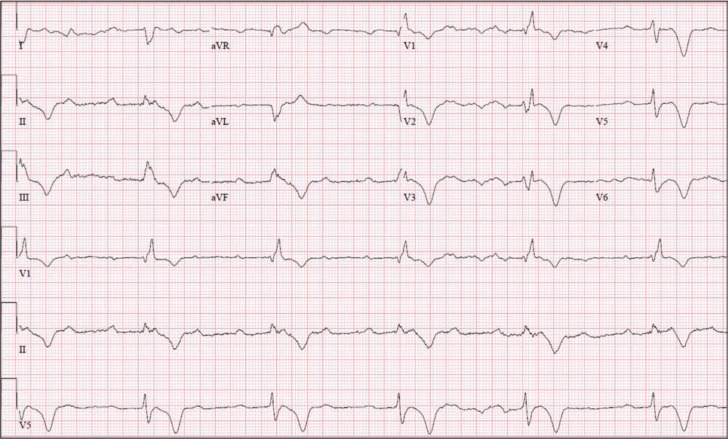
Electrocardiogram demonstrating complete heart block with a right bundle branch block pattern wide complex escape rhythm.

Our patient is a 76-year-old woman with a past medical history of complete infranodal heart block diagnosed in the year 2000 with an electrophysiology study. She underwent the implantation of a dual-chamber pacemaker, and in 2003 she presented with sudden cardiac arrest (in the setting of preserved left ventricular (LV) systolic function) that was attributed to long QT syndrome. She was upgraded to a dual-chamber implantable cardioverter-defibrillator (ICD) at that time. She did well for 12 years, until she presented in late September 2015 with RV lead fracture and total failure to capture. She was lightheaded with activity, and her electrocardiogram (ECG) demonstrated marked bradycardia and complete heart block **(Figure 1)**. She had a right bundle branch block (RBBB) pattern escape rhythm, and was monitored for 48 hours on telemetry prior to revising her lead as she was hemo-dynamically stable. Also, she had been developing progressive HF symptoms over the past three years, along with a cardiomyopathy. She was diagnosed with right breast cancer in 2012 and was treated with doxorubicin. An echocardiogram in early 2015 demonstrated that her LV ejection fraction had decreased to 35%. It was unclear if her cardiomyopathy may have been related to chemotherapy, or was due to pacing-induced cardiomyopathy.

**Figure 2:**
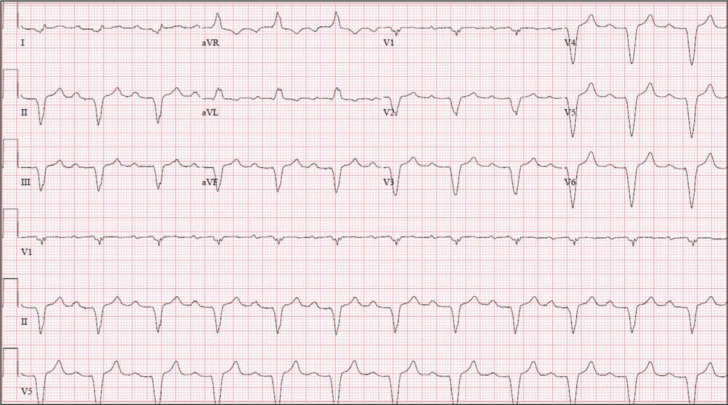
Ventricular paced electrocardiogram obtained prior to lead fracture. Note the wide left bundle branch block pattern QRS duration of approximately 170 ms. In this patient, the lead was found to be in the middle cardiac vein.

Given this patient’s complete heart block and worsening HF symptoms in the setting of a wide QRS RVP morphology, it was elected to upgrade her pacing system to a biventricular (BiV) ICD **(Figure 2)**. During the proce-coil was in the middle cardiac vein. Additionally, she had dure, a temporary pacing wire was initially placed from suboptimal coronary venous branches and LV pacing the right femoral vein. It was discovered that her old RV requiring very high outputs (4.5 V at 1 ms), resulting in left phrenic nerve capture. Her options included surgical placement of an epicardial LV lead or attempts at HBP. However, it was unclear whether HBP would help recruit conduction in a patient who had had long-standing infranodal block with no atrioventricular (AV) conduction for the past 15 years.

## Discussion

The BLOCK HF trial, reported by Curtis and colleagues, examined the efficacy of CRT in HF patients with high-grade AV block over RVP.^5^ Primary outcome (i.e. death from any cause, urgent care visit for HF requiring intravenous therapy, or an increase in LV end-systolic volume index ≥15%) occurred in 45.8% of CRT patients and 55.6% of RV-paced subjects. Although better outcomes were demonstrated with BiVP than with RVP, it should be noted that patients with BiVP did not fare well either with regards to high event rates. Also, the primary end-point was driven by a change in LV end-systolic volume index. LV stimulation can be achieved in patients with left bundle branch block (LBBB) and complete heart block by recruiting the native conduction system with HBP. Barba-Pinchardo et al. attempted HBP in candidates for CRT in whom LV stimulation via the coronary sinus was not achievable, and found that direct His-bundle pacing (DHBP) corrected conduction in 13 of the 16 patients (81%) selected.^6^ In nine patients, DHBP successfully resulted in definitive resynchronization with improvement in functional class and parameters of LV function. Similarly, Lustgarten et al. assessed DHBP in patients with standard indications for biventricular pacing, and noted that it resulted in a significantly narrower QRS as compared with native conduction and biventricular pacing (mean QRS duration: native 171 ms, DHBP 148 ms, and BiV 158 ms, p<0.0001).^7^ This finding of electrical resynchronization was revisited by the same group of researchers again in a crossover comparison study assessing the feasibility of, and the clinical response to, permanent HBP as an alternative to BiVP in CRT-indicated patients. It was found that HBP can elicit 6-month CRT clinical responses similar to those of BiVP.^8^ Therefore, HBP can be considered an alternative to CRT if the traditional method of LV resynchronization via the coronary sinus fails. Vijayaraman et al. demonstrated in a large series of patients that HBP can be successfully achieved in both nodal block and infranodal block contexts. The investigators were able to demonstrate chronic stable pacing thresholds with both selective and non-selective HBP (SHBP and NSHBP, respectively) in both AV nodal block and infranodal block patients, with minimal use of a backup RV pacing lead.^9^ Two types of responses can be seen with permanent HBP. SHBP occurs when the pacing stimulus to QRS onset is equal to the intrinsic HV interval with no fusion with local myocardium at the pacing output selected (i.e., a clear isoelectric segment in all 12 leads between pacing stimulus and QRS onset). NSHBP is defined as His-bundle capture with local myocardial fusion at the pacing output selected, and with the pacing stimulus to QRS onset being shorter than the intrinsic HV interval.

## Case revisited

**Figure 3:**
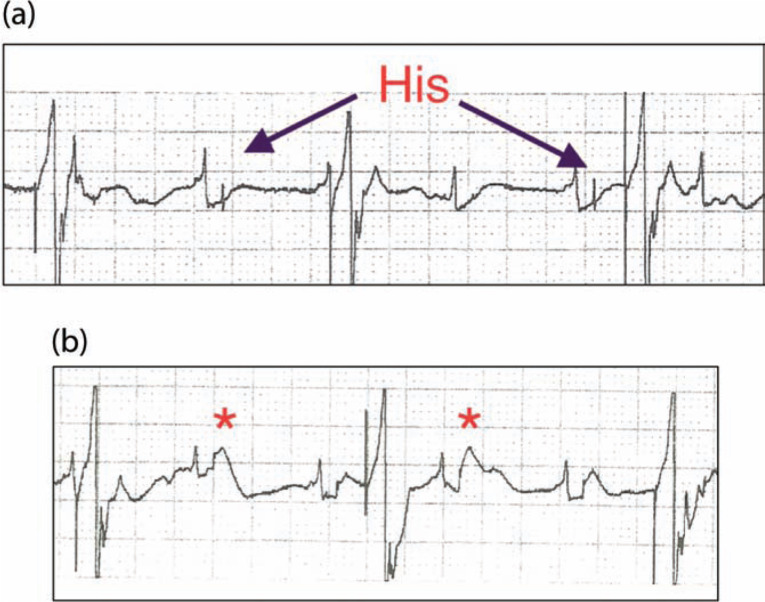
Intracardiac electrograms as recorded by the pacing lead through the pace-sense analyzer. The pacing lead is connected in a unipolar configuration, and back-up ventricular pacing is being performed through a temporary transvenous pacing wire. (a) Complete infranodal heart block with a paced ventricular rhythm. (b) Acute His bundle current of injury (asterisk) after the lead is screwed into the tissue.

**Figure 4:**
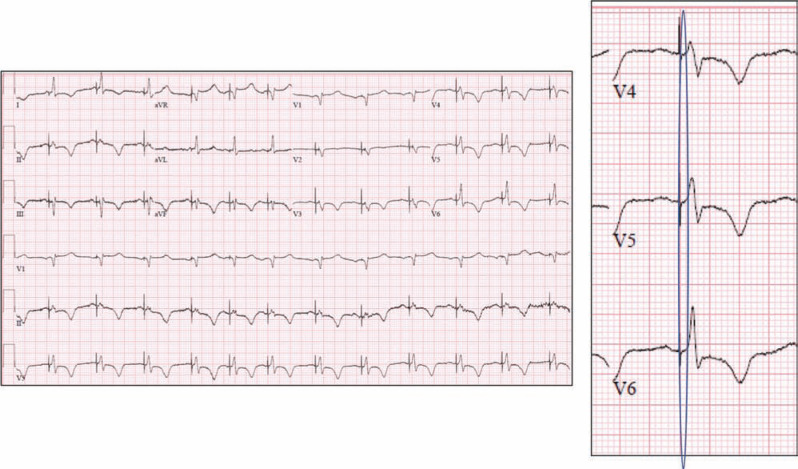
A 12-lead electrocardiogram (ECG) with non-selective His-bundle pacing (NSHBP) taken immediately after the procedure was completed. Note the deep T-wave inversions seen right after the His-bundle tissue is recruited with pacing, due to T-wave memory phenomenon. The T-wave inversions normalized by four weeks after the procedure. The magnified ECG shows leads V4 to V6, with the highlighted area showing local myocardial capture with no isoelectric segment between the pacing stimulus and QRS onset, confirming NSHBP. This ECG demonstrates that even in a patient with longstanding infra-nodal complete heart block (>15 years), permanent His-bundle tissue recruitment can occur with pacing at the site of block.

**Figure 5:**
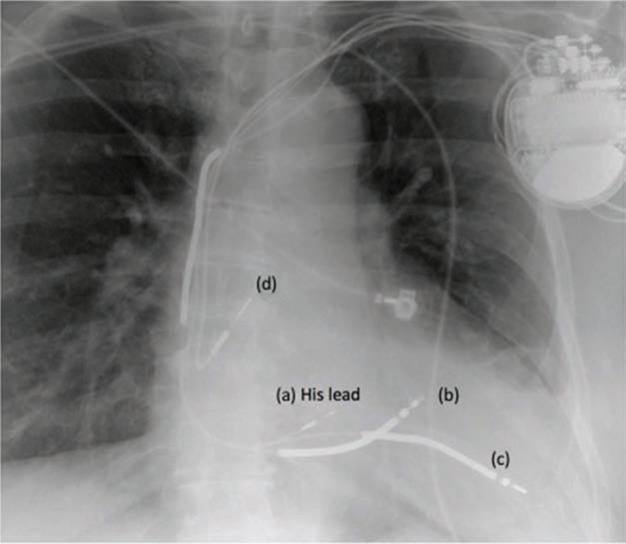
A chest X-ray taken post-procedure. (a) His-bundle pacing lead. (b) The new high voltage lead in the right ventricle. (c) The old high voltage lead in the middle cardiac vein. (d) A pacing lead in the right atrium.

It was elected to try HBP in this patient and, if it failed, the decision was made that she would be referred for surgical LV lead placement instead. His-bundle mapping was performed using a C315 His sheath and a 3830 pacing lead (Medtronic, Inc., Minneapolis, MN, USA). In a unipolar configuration, clear His-bundle electrograms were recorded, demonstrating complete infranodal block and, once the lead was screwed into place, His-bundle current of injury **(Figure 3a,b)**.^10^ In this case, NSHBP was achieved via reducing pacing output from 5 V at 1 ms to 1 V at 1 ms **(Figure 4)**. The His-bundle lead was connected into the LV port, and the RV lead (i.e., the new RV single coil ICD lead) was connected into the RV port **(Figure 5)**. LV to RV offset was programmed to 80 ms to promote HBP while minimizing the possible effects of fusion (that would result in functional non-capture). Her old RV ICD lead was capped. The patient reported significant improvement in her symptoms (from NYHA class II to class I), and a repeat cardiac electrocardiogram 3 months later demonstrated normalization of her LV systolic function. Owing to the rapid reversal of LV systolic function, it was felt that her cardiomyopathy was primarily related to pacing-induced cardiomyopathy.^11^ Seventeen months later, she remains symptom-free, with no functional limitations. Pacing thresholds remain at 0.5 V at 1 ms with SHBP and no fusion, and NSHBP at outputs > 1 Vat 1 ms.

## The evidence behind His-bundle pacing

The bundle of His is an important part of the conduction system that transmits impulses from the AV node to the ventricles. The AV node and the bundle of His constitute the AV junction. The bundle of His then branches into the right and left bundle branches, which divide into fascicles, giving rise to the Purkinje fibers. These specialized cardiac myocytes are named after the Swiss anatomist and cardiologist Wilhem His, Jr., who first discovered them in 1893.^12^ Scherlag and colleagues published their technique for recording His-bundle activity in humans in 1969.^13^ Functional longitudinal dissociation of the His-bundle was first proposed by Kaufman and Rothberger in 1919.^14^ Predestined fibers within the His bundle selectively conducted to the individual bundle branches, and these fibers originated within the proximal portions of the common bundle. This concept was demonstrated in humans by Narula in 1977.^15^ Patients with LBBB and baseline prolonged HV intervals were paced slightly distal to the proximal His-bundle, resulting in a narrowing of the QRS.

In 2000, Deshmukh et al. demonstrated the first clinical trial with permanent HBP in a group of 18 patients with a history of chronic AF, dilated cardiomyopathy and normal ventricular activation.^16^ Following AV nodal ablation, HBP was successful in 12 of these patients, and resulted in an improvement in LV function in almost all of the studied patients. Since then, several investigators around the world have published their own experiences with permanent HBP, and the field has gradually progressed since then.

Barba-Pichardo et al. attempted HBP in selected patients referred for pacemaker implantation, and found that HBP corrected conduction abnormalities in 73% of patients (32% of whom did not undergo permanent HBP due to high thresholds during initial testing).^17^ HBP has been shown to correct bundle branch blocks in the presence of complete AV block (considered to be infra-Hisian heart block), resulting in a normal QRS complex. In addition to normalizing bundle branch blocks, several other studies considered the hemodynamic and clinical benefits of HBP over right ventricular apical pacing (RVAP). Kronborg et al. showed that stable direct HBP or para-Hisian pacing is feasible in 85% of patients with narrow QRS and high-grade AV block, and leads to normalization of the ventricular activation pattern with statistically significantly less LV dyssynchrony as compared with RVP.^18^ Catanzariti et al. also examined HBP effects acutely and found that it prevents inter- and intraventricular asynchrony either on the same day as the procedure, or on the following day.^19^ These beneficial effects of improved ventricular contractile performance persisted after 2 years of follow-up. This reduced asynchronous pacing induced LV ejection fraction depression and mitral regurgitation in comparison with RVAP.^20^

Similarly, Zanon et al. conducted a crossover mid-term study in which patients underwent 3 months of DHBP, and then three months of RVAP. Myocardial perfusion was found to be significantly better during DHBP than during RVAP.^21^ In a study by Pastore et al., 37 patients with normal cardiac function had permanent HBP with an apical RV backup lead, and underwent 3 months of HBP followed by 3 months of RVAP.^22^ Switching from HBP to RVAP resulted in a significant increase in systolic and diastolic electromechanical delays and consequent inter- and intraventricular dyssynchrony. Finally, one of the largest studies on HBP was published by Sharma et al., and its findings supported the clinical benefits of HBP previously examined in smaller studies; namely, HF hospitalization was reduced in the HBP group compared with the RVP group, with no difference in mortality noted between the two groups.^23^ However, it should be noted that this study was not powered to detect mortality benefit. Selective studies comparing HBP and RV pacing are summarized in **Table 1**.

When it comes to the safety of HBP, Vijayaraman et al. noted that catheter manipulation during HBP caused injury to the His-bundle in 7.8% of patients undergoing permanent HBP with resultant RBBB, in most cases. However, complete spontaneous resolution of the RBBB occurred in most cases, and RBBB itself persisted in 2.5% of the cases (all normalized with HBP).^24^ Furthermore, Sharma et al. found that permanent HBP without a mapping catheter or a backup RV lead was successfully achieved in 80% of patients.^25^ Pacing thresholds were higher and fluoroscopy times were similar in these individuals to those of the RVP group. Furthermore, no increased risks were noted when the procedure was performed by experienced operators. The higher pacing threshold is relatively problematic depending on the type of HBP that is pursued. As described earlier, SHBP and SHHBP can be encountered. Given that NSHBP is capturing myocardium in addition to the His-bundle, it may be preferred in patients with complete heart block, where a backup RV lead is not utilized. NSHBP has also been associated with lower pacing thresholds than SHBP. No statistically significant differences in clinical outcomes were noted between the two types of pacing in the study conducted by Catanzariti et al., but there still needs to be further investigation completed via larger randomized controlled trials prior to drawing any final conclusions.^19^

Several issues unique to HBP also need to be considered carefully. These include both undersensing and over-sensing issues, local atrial capture from HBP, elevated pacing thresholds, and increased battery drain because of inherently higher pacing thresholds than RVP. With improvements in lead design and pacemaker technology relating to permanent HBP, it is likely that we will soon have better device systems that successfully address all of these issues.

## Conclusions

Although the observations initially made by Deshmukh et al. represent an important step towards achieving true physiological pacing, the adoption of HBP has been slow due to perceptions that permanent HBP is technically challenging, and may not be possible in heart block and bundle branch block patients. However, in recent years, these myths have been broken, and the electrophysiology community has begun to realize the true potential of this form of pacing. With continued technological advances and the demonstration of clinical benefits through large-scale randomized trials, permanent HBP is likely to find an important role in permanent cardiac pacing.

## Figures and Tables

**Table 1:** Electrophysiological, Hemodynamic and Clinical Differences Between HBP and RVP

Study	Research Design	N	Results	Limitations
Kronborg et al.^17^ (2012)	Comparative study	38	Mean QRS duration was 153 ± 12 ms with HBP and 161 ± 15 ms with apical pacing.	Small sample size.
Catanzariti et al.^18^ (2006)	Comparative study	23	Decreased indexes of ventricular dyssynchrony, mitral regurgitation and improvement of systolic ventricular function Tei index with HBP. No statistically significant differences between DHBP and para-Hisian pacing.	Only acute variations in ventricular function were assessed, and the long-term clinical benefit (on the same day as the implantation procedure or the following day) remained unclear. A two-dimensional imaging technique assessing for basal dyssynchrony was used.
Catanzariti et al.^19^ (2012)	Prospective cohort study	26	Decreased EF (50.1 ± 8.8% versus 57.3 ± 8.5%, p<0.001); increased MR (22.5 ± 10.9% versus 16.3 ± 12.4%; p = 0.018) and worsening interventricular delay (33.4 ± 19.5 ms versus 7.1 ± 4.7 ms, p = 0.003) with RVAP, compared with HBP.	Small sample size. Non-randomized observational study type.
Zanon et al.^20^ (2008)	Crossover study	12	Better perfusion score with DHBP than with RVAP (0.44 ± 0.5 versus 0.71 ± 0.53, respectively; p = 0.011).	Small sample size. No pre-implantation coronary angiography due to the urgent indication for permanent pacing.
Pastore et al.^21^ (2014)	Crossover study	37	Increased S–D EMD (p = 0.001) and intra-LV dyssynchrony (p = 0.001); increased LV isovolumetric contraction time (p = 0.001) and LV isovolumetric relaxation time (p = 0.05); and decreased LV ejection time (p = 0.033) with RVAP, in comparison with HBP.	Non-randomized crossover study.
Sharma et al.^22^ (2015)	Prospective cohort study	173	Similar fluoroscopy times (12.7 ± 8 min versus 10 ± 14 min; median 9.1 versus 6.4 min; p = 0.14) and higher stable pacing thresholds were observed in the HBP group than in the RVP group (1.35 ± 0.9 V versus 0.6 ± 0.5 V at 0.5 ms; p<0.001). Decreased HF hospitalization in the HBP group as compared with the RVP group (2% versus 15%; p = 0.02) was observed, with no difference in mortality between the two groups (13% in the HBP group versus 18% in the RVP group; p = 0.45).	Non-randomized observational study type.

DHBP, direct His-bundle pacing; EF, ejection fraction; HF, heart failure; HBP, His-bundle pacing; LV, left ventricular; MR, mitral regurgitation; S-D EMD, systolic-diastolic electromechanical delay; RVAP, right ventricular apical pacing; RVP, right ventricular pacing.
